# Effect of cannabis use on blood levels of brain‐derived neurotrophic factor (BDNF) and nerve growth factor (NGF): A systematic review and meta‐analysis

**DOI:** 10.1002/brb3.3340

**Published:** 2023-12-31

**Authors:** Arman Shafiee, Mohammad Ali Rafiei, Kyana Jafarabady, Alireza Eskandari, Faeze Soltani Abhari, Mohammad Amin Sattari, Mohammad Javad Amini, Mahmood Bakhtiyari

**Affiliations:** ^1^ Department of Psychiatry and Mental Health Alborz University of Medical Sciences Karaj Iran; ^2^ Student Research Committee, School of Medicine Alborz University of Medical Sciences Karaj Iran; ^3^ School of Medicine Shahid Beheshti University of Medical Sciences Tehran Iran; ^4^ School of Pharmacy Islamic Azad University of Medical Sciences Tehran Iran

**Keywords:** brain‐derived neurotrophic factor, cannabis, nerve growth factor, substance use disorder

## Abstract

**Background:**

The impact of cannabis uses on blood levels of brain‐derived neurotrophic factor (BDNF) and nerve growth factor (NGF) remains uncertain, with conflicting findings reported in the literature. BDNF and NGF both are essential proteins for neuron's growth, and their dysregulation is seen in various mental disorders. This study aims to evaluate the relationship between cannabis usage and BDNF and NGF levels due to their potential implications for mental health.

**Methods:**

A comprehensive search of electronic databases was performed using appropriate MeSH terms and keywords. Inclusion criteria comprised human studies investigating the relationship between cannabis use and BDNF and NGF levels.

**Results:**

A total of 11 studies met the inclusion criteria and were included. The pooled analysis revealed a nonsignificant association between cannabis use and dysregulated blood levels of BDNF (random‐effects model, standardized mean differences [SMD] = .26, 95% CI −.34 to .76, *p* = .40). The results of our subgroup analysis based on BDNF source showed a nonsignificant between‐group difference. For NGF levels, four studies were included, the pooled analysis revealed a nonsignificant association between cannabis use and dysregulated blood levels of NGF (random‐effects model, SMD = −.60, 95% CI −1.43 to –.23, *p* = .16). In both analyses, high heterogeneity was observed among the included studies which is a notable limitation to current meta‐analysis.

**Conclusion:**

This systematic review highlights the need for further research to elucidate the relationship between cannabis use and these neurotrophic factors. A better understanding of these associations can contribute to our knowledge of the neurobiological effects of cannabis and inform potential implications for mental health, cognitive function, and neurodegenerative disorders.

## Graphical abstract

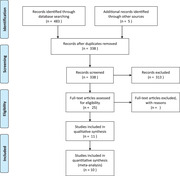

## 1 INTRODUCTION

Cannabis, commonly known as marijuana, is one of the most widely used psychoactive substances globally (Lafaye et al., 2017). Its use has increased significantly in recent years, both for recreational purposes and for the management of various medical conditions (Han & Palamar, 2020). The active compounds in cannabis, particularly delta‐9‐tetrahydrocannabinol (THC) and cannabidiol, have been extensively studied for their effects on the central nervous system and their potential therapeutic properties (Černe, 2020). However, the impact of cannabis use on the levels of neurotrophic factors in the blood, specifically brain‐derived neurotrophic factor (BDNF) and nerve growth factor (NGF), remains a topic of considerable interest and debate.

BDNF and NGF are essential proteins involved in the growth, development, and survival of neurons in the brain and peripheral nervous system (Binder & Scharfman, 2004). BDNF plays a crucial role in synaptic plasticity, neurogenesis, and neuronal survival, whereas NGF promotes the growth, maintenance, and function of sensory and sympathetic neurons (Andres & Bradshaw, 1980; Autry & Monteggia, 2012). Alterations in the levels of these neurotrophic factors have been implicated in various neurological and psychiatric disorders, including depression, anxiety, neurodegenerative diseases, and cognitive impairments (Dou et al., 2022; Salles et al., 2017).

Preclinical studies have suggested that cannabis and its constituents may modulate BDNF and NGF levels, thereby influencing neuroplasticity and neuronal survival (). However, the findings from human studies investigating the effects of cannabis use on BDNF and NGF levels have been inconsistent and inconclusive. Some studies have reported a decrease in BDNF and NGF levels among cannabis users, whereas others have found no significant association or even contradictory results (10) (Angelucci et al., 2008).

The impact of cannabis use on BDNF and NGF levels due to their potential implications for mental health, cognitive function, and neurodegenerative disorders should be investigated. A systematic review and meta‐analysis can provide a comprehensive and rigorous assessment of the available evidence, helping to elucidate the relationship between cannabis use and these neurotrophic factors. Therefore, the aim of this study is to synthesize the existing literature and evaluate the effect of cannabis use on blood levels of BDNF and NGF.

## 2 METHODS

### 2.1 Research question and objective

The research question guiding this systematic review was as follows: “What is the effect of cannabis use on blood levels of Brain‐Derived Neurotrophic Factor (BDNF) and Nerve Growth Factor (NGF)?”

### 2.2 Study protocol and search strategy

The protocol for this systematic review and meta‐analysis was developed a priori and registered in PROSPERO (CRD42023433718). The preferred reporting items for systematic reviews and meta‐analyses guidelines were followed to ensure the transparency and rigor of the review process (Page et al., 2021).

A comprehensive search strategy was developed in consultation with a research librarian to identify relevant studies. Electronic databases, including PubMed, Embase, Scopus, and Web of Sciences, were searched from inception to October 26, 2023. The following search term was applied in each database: (cannabis OR marijuana OR cannabis use OR marijuana use OR THC OR Δ9‐THC) AND (blood OR serum OR plasma OR circulating) AND (BDNF OR NGF). The search strategy also involved manual searching of reference lists of relevant articles and review papers to identify additional studies. No language or publication date restrictions were applied during the search process.

### 2.3 Study selection

Two independent reviewers conducted the initial screening of identified articles based on the titles and abstracts. Full‐text articles were obtained for potentially relevant studies. Any discrepancies during the selection process were resolved through discussion or consultation with a third reviewer.

Inclusion criteria: Studies investigating the effect of cannabis use on blood levels of BDNF and NGF. Human studies. Original research articles published in peer‐reviewed journals.

Exclusion criteria: Animal studies, reviews, case reports, and conference abstracts. Studies not reporting blood levels of BDNF or NGF. Studies not directly assessing the relationship between cannabis use and BDNF or NGF levels.

### 2.4 Data extraction and quality assessment

Data extraction was performed independently by two reviewers using a standardized data extraction form. The following information was extracted from each included study: author(s), year of publication, study design, sample characteristics, cannabis exposure duration, BDNF and NGF measurement methods, and the quality of the included studies.

The methodological quality and risk of bias assessment of included studies were evaluated using appropriate tools, such as the Newcastle–Ottawa scale or the Cochrane Risk of Bias Tool, depending on the study design. Any disagreements were resolved through consensus or by consulting a third reviewer (Luchini et al., 2017).

### 2.5 Data synthesis and meta‐analysis

A meta‐analysis was conducted using RevMan 5.4 to calculate pooled effect estimates (standardized mean differences [SMD]) and their corresponding 95% confidence intervals. Heterogeneity among studies was assessed using statistical tests (e.g., Cochran's *Q* test, *I* ^2^ statistic). Subgroup analysis was performed to explore potential sources of heterogeneity. Publication bias was assessed using funnel plots and statistical tests (e.g., Egger's test) if a sufficient number of studies were included (*n* > 10).

## 3 RESULTS

### 3.1 Study selection

**FIGURE 1:**
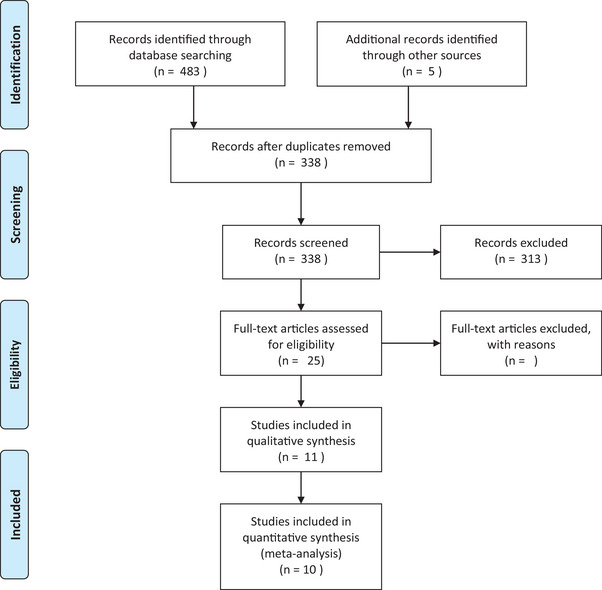
Preferred reporting items for systematic reviews and meta‐analyses (PRISMA) flow diagram.

The initial search yielded a total of 337 articles after removing duplicates. After screening the titles and abstracts, 25 articles were selected for full‐text review. Following the full‐text review, 11 studies met the inclusion criteria and were included in the quantitative synthesis. A flow diagram illustrating the study selection process is presented in Figure 1 (Angelucci et al., 2008; Bayazit et al., 2019; Chagas et al., 2014; D'Souza et al., 2009; Jockers‐Scherubl et al., 2004, 2006; Lisano et al., 2020; Miguez et al., 2019; Szabo et al., 2020; Toll et al., 2020; Yazici et al., 2022).

### 3.2 Study characteristics and quality assessment

The included studies were published between 2004 and 2022 (Table 1). All studies were case–control with regard to study design. The sample sizes ranged from 23 to 643 participants.

**TABLE 1:** Characteristics of the included studies.

Author	Year	Country	Type of study	Population	Number of participants (current cannabis//control)	Age (mean)	Male (%)	Source of sampling	NGF and BDNF measurement tool	Type of BDNF	Quality
Angelucci, F.	2008	Italy	Case–control	Chronic cannabis‐users	46 (26/20)	Cannabis = 27.30 ± 5.64/control = 28.25 ± 4.84	52	Serum	Sandwich ELISA	Mature	Good
Chagas, M. H. N.	2014	Brazil	Case–control	Cannabidiol treatment	21 (14/7)	51–82	71	Plasma	ELISA	N.R.	Fair
D'Souza, D.	2009	USA	Case–control	Light users of cannabis	23 (9/14)	24.6 (±6.56)	86	Serum	ELISA	N.R.	Good
Jockers‐Scherubl, M. C.	2004	Germany	Case–control	Chronic cannabis‐users	386 (46/172)	18−62	56	Serum	ELISA	N.R.	Fair
Jockers‐Scherubl, M. C.	2006	Germany	Case–control	Chronic cannabis‐users	195 (48/24/51)	19–60	98	Serum	ELISA	N.R.	Fair
Lisano, J. K.	2020	USA	Case–control	Physically active individuals with chronic cannabis‐users	30 (15/15)	23.4 ± 4.4	75	Serum	ELISA	N.R.	Good
Miguez, M. J.	2019	USA	Case–control	Mixed	500 (200/300)	16.5 ± 1.4/non = 14.5 ± 2.2	45–48	Plasma	MILLIPLEX	Mature	Fair
Szabo, A.	2020	Norway	Case–control	Mixed	643 (97/546)	18–65	47	Plasma	ELISA	N.R.	Good
Yazici, A. B.	2022	Turkey	Case–control	Chronic cannabis‐users	54 (27/27)	29.62 ± 6.12/non = 30.70 ± 7.05	100	Serum	ELISA	N.R.	Fair
Bayazit	2019	USA	Case–control	DSM‐IV diagnosed cannabis users	90 (45/45)	25 ± 8/non: 25 ± 8	100	Serum	ELISA	Total	Good
Toll, A	2020	Spain	Cross sectional observational study	Patients with first episode psychos with mixed users and healthy volunteers	127 (73/54)	21.91 and 26.29	67.72	Serum	Sandwich ELISA	Mature	Good

Abbreviations: BDNF, brain‐derived neurotrophic factor; NGF, nerve growth factor.

The participants in the included studies were predominantly adults (age range: 18–65 years) with various cannabis use patterns. Most studies focused on chronic cannabis users, one study was conducted on light users, three studies were conducted on both chronic and light users, and one examined medical cannabis users. Regarding the study population, there were four studies conducted on patients with past neuropsychiatric conditions (Chagas et al., 2014; Jockers‐Scherubl et al., 2004, 2006; Szabo et al., 2020).

The key findings from each of the included articles are summarized in the Table 2. Notably, the majority of these studies did not identify a significant relationship between cannabis consumption and BDNF levels. Eleven studies were included in our analysis based on the established criteria. In most instances, individuals did not present any concurrent mental illnesses aside from cannabis use disorder. However, in two articles, schizophrenia was observed in a subset of participants. Additionally, one article reported the occurrence of first‐episode psychosis, the other featured individual with bipolar disorder (BD), and yet another study focused on individuals with Parkinson's disease (PD). When examining cannabis consumption patterns across the studies, several common patterns emerged. Chronic consumption was prevalent, with most individuals consuming at least 0.5 g of cannabis per day for a duration ranging from 6 months to 2 years. The specific consumption patterns for each study are detailed in Table 2.

**TABLE 2:** Main results of the included studies.

Author/year	Cannabis use pattern	Past psychiatric history	Main finding
Angelucci, F./2008	The study reports chronic cannabis consumption at a mean age of 16.5 ± 2.65 and a mean duration of consumption at the date of investigation of 10.73 ± 5.22 years. The frequency of cannabis use was 5.42 times/week (SD 2.04)	No additional psychiatric or somatic disorder	Study investigated the potential relationship between chronic cannabis consumption and alterations in the serum levels of the neurotrophins NGF and BDNF, which are important molecules for neuronal survival and function in the CNS NGF serum levels were significantly reduced in cannabis abusers compared to healthy subjects, whereas BDNF serum levels remained unchanged
Chagas, M. H. N./2014	Patients were given either a placebo or different doses of CBD (75 or 300 mg/day) for 6 weeks. The baseline assessment was repeated in the last week of the study to evaluate the effects of CBD on the patients	They have no history of past psychiatric history expect Parkinson's disease	The study found no differences between the groups treated with CBD and placebo in respect to BDNF levels at baseline and after 6 weeks, nor in the different measures using H1‐MRS (NAA/Cre)
D'Souza, D./2009	Light user (not mentioned exactly)	Not reported	The text suggests that cannabis exposure may contribute to the development of schizophrenia or psychotic disorders, but it is not sufficient to cause these conditions alone. The study found no significant differences between the groups treated with CBD and placebo in respect to BDNF levels at baseline and after 6 weeks, and no significant side effects were recorded
Jockers‐Scherubl, M. C./2004	It is characterized by a mean consumption of at least 0.5 g of cannabis per day for at least 2 years	No additional psychiatric or somatic disorder	The text suggests that raised BDNF serum levels are not related to schizophrenia and/or substance abuse itself but may reflect a cannabis‐related idiosyncratic damage of the schizophrenic brain
Jockers‐Scherubl, M. C./2006	Long‐term (chronic) cannabis consuming control persons with a previous cannabis intake of at least 0.5 g/day for at least 2 years and no additional psychiatric or somatic disorder	Just schizophrenia in a group	The study found that there was no significant difference in serum NGF concentrations between treated and largely recovered schizophrenic patients, regardless of previous cannabis, or other substance abuse before the first episode of the disease
Lisano, J. K./2020	Chronic cannabis use is defined as the regular use of cannabis products at least once per week for the last 6 months	Not reported	Plasma BDNF was significantly lower in physically active cannabis users compared to nonusers
Miguez, M. J./2019	“Once or twice” substance use, monthly use, weekly/daily	No additional psychiatric or somatic disorder	The search results suggest that younger adolescents who experiment with marijuana and engage in moderate use have increased BDNF levels, whereas older adolescents who escalate their use have a steeper increase in endogenous BDNF levels
Szabo, A./2020	“Cannabis use” was Defined as any use in the last 6 months prior to blood sampling	Bipolar disorder patient and schizophrenia	The search results suggest that cannabis self‐administration is associated with higher sgp130 levels in schizophrenia but not in bipolar disorder, and this phenomenon is independent of the modulation of peripheral immune cells
Yazici, A. B./2022	Cannabis use day one and seventh	No additional psychiatric or somatic disorder	The search results suggest that there is a relationship between verbal aggression and BDNF and NGF serum levels in schizophrenia The study found that there was no significant difference
Bayazit/2019	Cannabis‐dependent patients, diagnosed according to DSM‐IV‐TR	No additional psychiatric or somatic disorder	Results suggest that patients with cannabis dependence have significantly increased BDNF, ceruloplasmin, and lipid hydroperoxide levels and decreased free thiol levels
Toll, A/2020	(None, low use, or high use) established in the cannabis experience questionnaire	Patients with first episode psychos with mixed users	The search results suggest that there is no relationship between cannabis use and BDNF levels in HV, but there is a different association between cannabis use and BDNF levels in FEP patients, particularly with high doses of cannabis. Additionally, cannabis use is significantly associated with tobacco use, so that high cannabis users are also high tobacco users

Abbreviations: BDNF, brain‐derived neurotrophic factor; CBD, cannabidiol; CNS, central nervous system; FEP, first‐episode psychosis; HV, healthy volunteers; NGF, nerve growth factor.

Overall, the quality of the included studies varied, with some studies demonstrating a low risk of bias, whereas others had limitations in selection and exposure domains (see Supporting Information for detailed assessment).

### 3.3 Effect of cannabis use on BDNF levels

**FIGURE 2:**
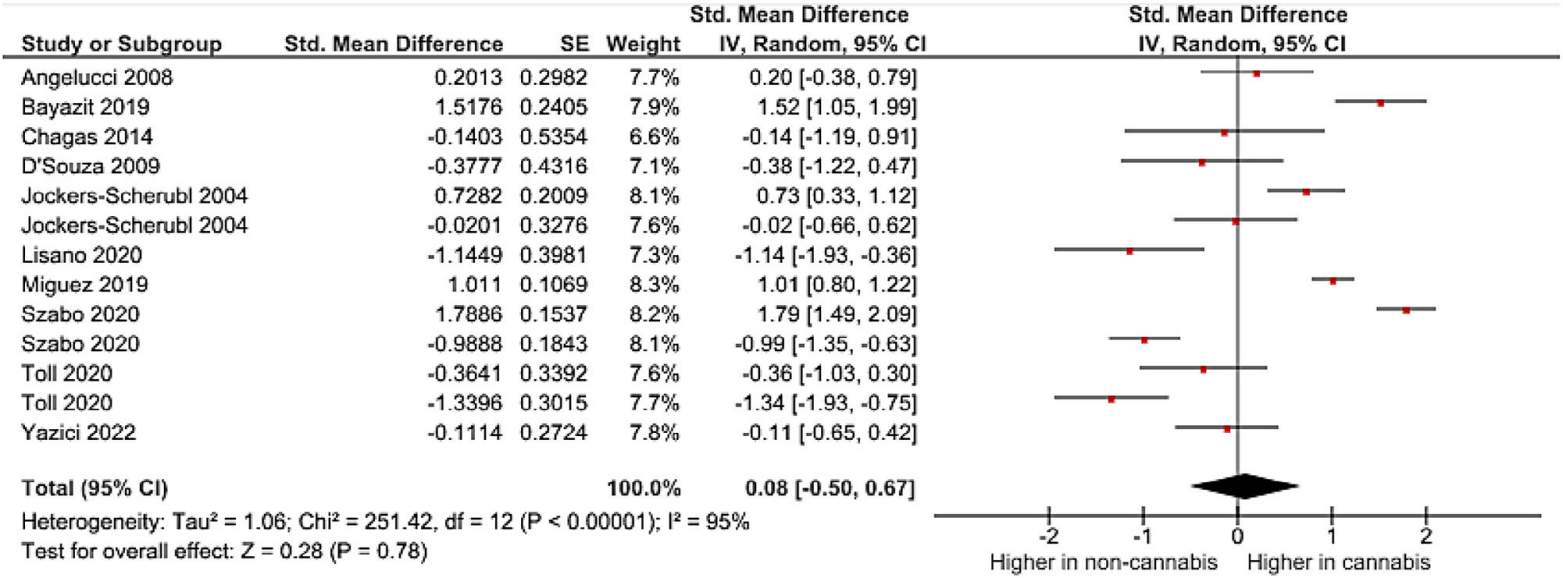
Results of meta‐analysis for the level of brain‐derived neurotrophic factor (BDNF) levels in cannabis users.

**FIGURE 3:**
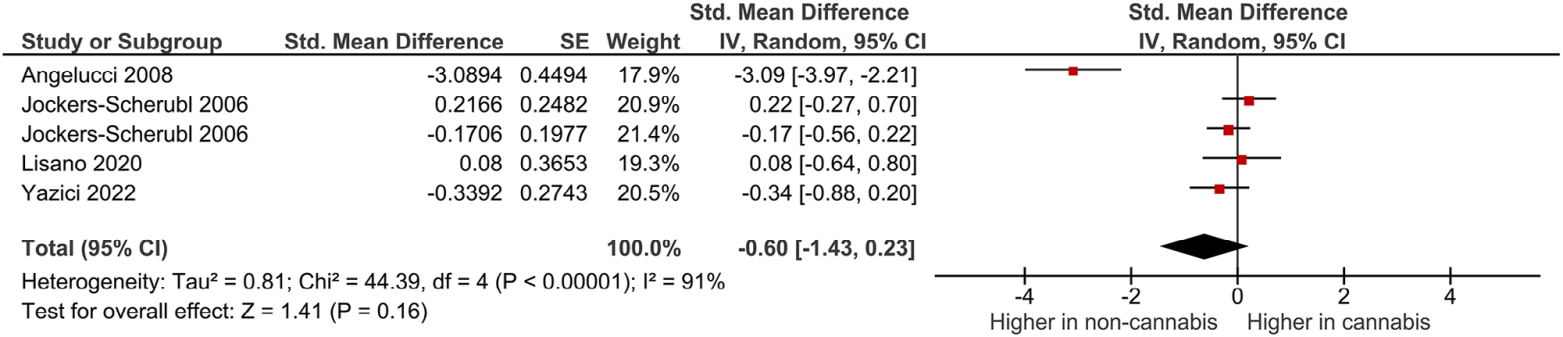
Results of meta‐analysis for the level of nerve growth factor (NGF) levels in cannabis users.

**FIGURE 4:**
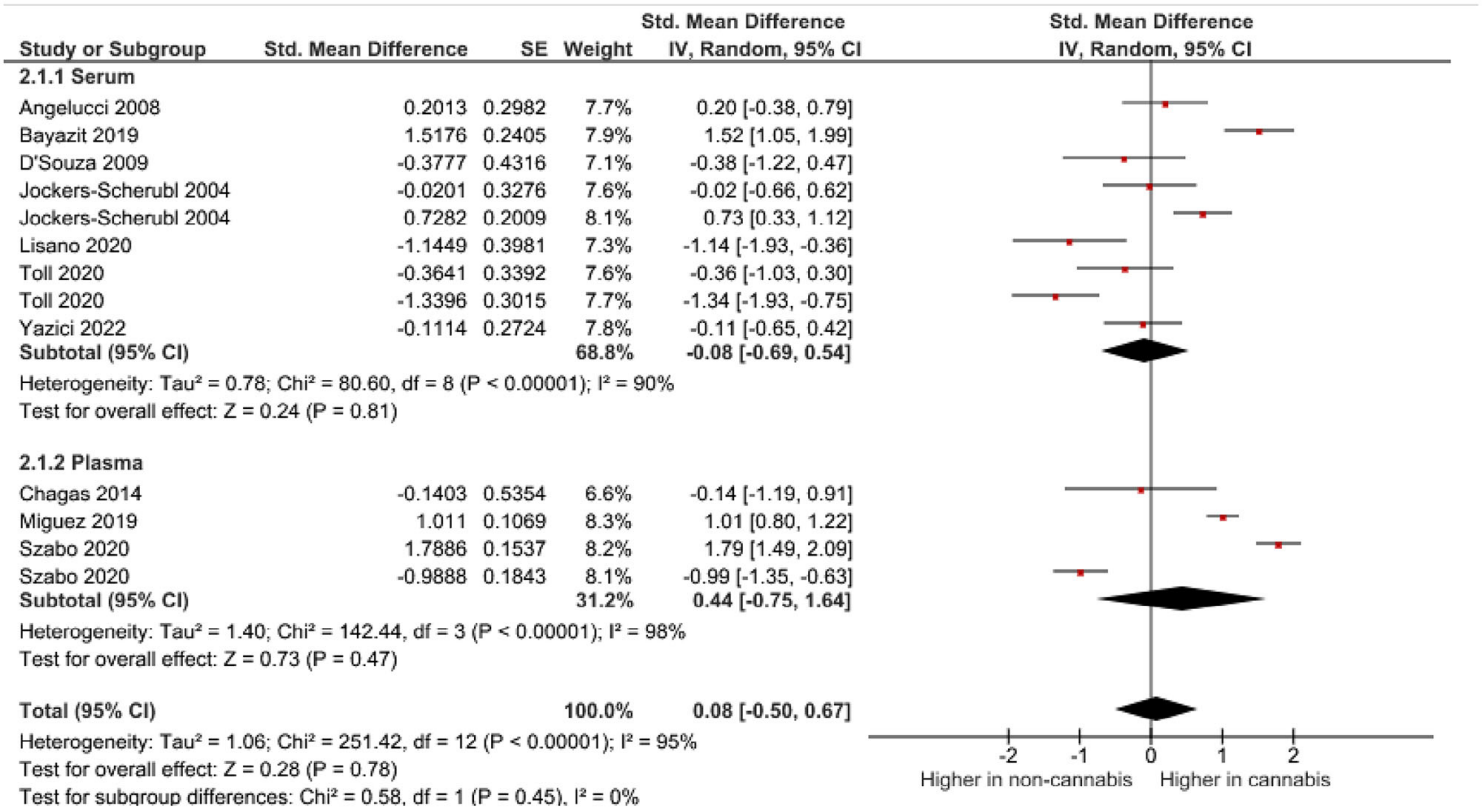
Subgroup analysis based on source of brain‐derived neurotrophic factor (BDNF).

**FIGURE 5:**
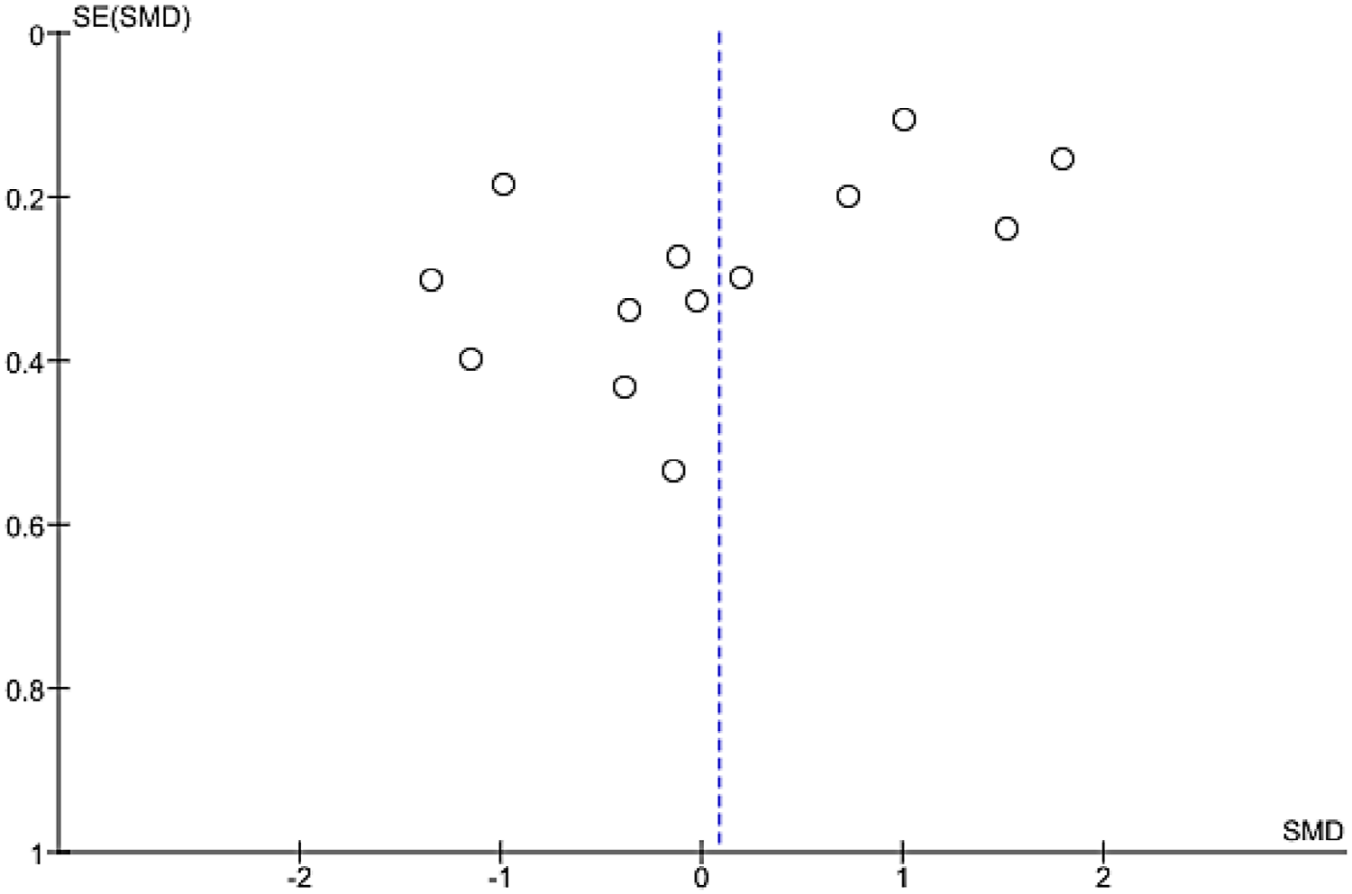
Funnel plot.

A meta‐analysis was performed on 10 studies that reported quantitative data on BDNF levels in cannabis users. The pooled analysis revealed a nonsignificant association between cannabis use and dysregulated blood levels of BDNF (random‐effects model, SMD = .26, 95% CI −.34 to .76, *p* = .40) (Figure 2 and Figure 3). High heterogeneity was observed among the included studies (*I* ^2^ = 95%). As there was an adequate number of effect sizes in the analysis (*n* ≥ 10), subgroup analysis was performed. The results of our subgroup analysis based on BDNF source showed a nonsignificant between‐group difference (Figure 4). There were no possible sources of publication bias based on the funnel plot and results of Egger's test (*p* = .12) (Figure 5).

### 3.4 Effect of cannabis use on NGF levels

A meta‐analysis was performed on four studies that reported quantitative data on NGF levels in cannabis users. The pooled analysis revealed a nonsignificant association between cannabis use and dysregulated blood levels of NGF (random‐effects model, SMD = −.60, 95% CI −1.43 to .23, *p* = .16) (Figure 3 ). High heterogeneity was observed among the included studies (*I* ^2^ = 91%). Publication bias could not be adequately assessed due to the limited number of studies included in the quantitative synthesis.

## 4 DISCUSSION

To the best of our knowledge, this is the first systematic review and meta‐analysis investigating the association between cannabis use and BDNF and NGF blood levels. In this analysis, we found no significant relationship between the dysregulation of BDNF or NGF levels and cannabis use. The nonsignificant relationship between BDNF levels and the source of sampling was consistent with the findings reported by Mohanraj et al. (2023). In addition to our meta‐analysis of the included studies, it is imperative to approach the interpretation of the nonsignificant effect of cannabis on BDNF levels with caution. This caution is warranted due to the limited agreement among the studies included in the analysis, which stems from variations in cannabis use patterns among subjects, discrepancies in the biofluid analyzed (serum vs. plasma), and differences in sample collection methods. Consequently, conducting a feasible meta‐analysis becomes challenging. Given the notable lack of consensus among the analyzed studies, we provided the qualitative review of the current state of knowledge rather than pursuing a meta‐analysis of neurotrophin content in cannabis users. Furthermore, we included three additional studies in our systematic review and also investigated the effect of cannabis on NGF in addition to BDNF levels. Several factors, including clotting duration, temperature, delay in blood sample centrifugation, and use of anticoagulants, may influence the measurement of BDNF both in plasma and serum (Amadio et al., 2017; Begliuomini et al., 2007). Plasma BDNF is unstable compared to serum BDNF (Kishino et al., 2001; Polyakova et al., 2017; Tsuchimine et al., 2014). In contrast, serum BDNF level may be subject to the BDNF released from platelets (Fujimura et al., 2002). These limitations may affect the results of included studies and hence our analysis. Evidence suggests that BDNF level is different among men and women (Lommatzsch et al., 2005; Piancatelli et al., 2022). Moreover, sex hormones and menstrual periods are considered factors that may alternate the BDNF level and function (Begliuomini et al., 2007; Chan & Ye, 2017; Pluchino et al., 2013). Thus, we should note the impact of the higher percentage of males in most included studies on the level of BDNF in our analysis results. Conducting studies that report both serum and plasma BDNF levels simultaneously and separately in men and women would be elucidative in this regard. A previous meta‐analysis by Green et al. (2011) found that BDNF level is decreased in both drug‐naïve and drug‐exposed patients with schizophrenia. Another meta‐analysis by Cui et al. (2012) reported the same results but evaluated this finding as low‐quality evidence. The available evidence supports that cannabis use is a risk factor for developing schizophrenia, although the attributive mechanism is not thoroughly understood (Arseneault et al., 2004; D'Souza et al., 2009; Henquet et al., 2005; Semple et al., 2005). A previous longitudinal study by Miguez et al. showed that group‐based trajectory modeling identified four distinct groups based on marijuana use patterns: naive individuals, starters, chronic users, and experimenting/quitters. Those who initiated marijuana use had similar levels of m‐BDNF compared to the control group. However, after adjusting for confounding factors, it was observed that younger adolescents showed an increase in BDNF levels during experimentation and moderate marijuana use compared to controls. Older adolescents had a steeper increase in endogenous BDNF levels, particularly during escalating marijuana use (Miguez et al., 2019). Interestingly, we found no significant relationship in BDNF peripheral level among healthy cannabis users compared to no‐users. This outcome may imply that a decrease in BDNF levels may not be the underlying cause of schizophrenia development in cannabis users. However, it is important to acknowledge that the lack of a follow‐up for subjects in the studies included weakens this interpretation. Therefore, additional research studies, particularly longitudinal studies, are essential to investigate this matter further.

Medications used for schizophrenia treatment like antipsychotics show an increase in BDNF levels in patients with schizophrenia (Fernandes et al., 2015; Mosiołek et al., 2023), and cannabis use is thought to be associated with worse outcomes in patients with psychosis and schizophrenia (Bersani et al., 2002; Schoeler et al., 2016; Zammit et al., 2008). Moreover, higher levels of BDNF are positively correlated with a better course of disease in patients with schizophrenia (Asevedo et al., 2013; Carlino et al., 2011; Zhang et al., 2012). Surprisingly, our findings indicate that schizophrenia patients who use cannabis have elevated BDNF levels as opposed to schizophrenia patients who do not use cannabis. There are a few potential explanations for this unexpected outcome, such as a potential variance in how cannabis affects patients who are taking antipsychotic medication or a limited number of studies analyzed in this specific subgroup. In addition, Segal‐Gavish et al.’s (2017) research provides insight into an alternative explanation, demonstrating that exposure to THC may cause a surge in BDNF expression, which acts as a regulatory response against cannabis use. To gain a better understanding of the relationship between cannabis use and BDNF levels in patients with schizophrenia, further research is necessary.

It is suggested that BDNF plays an important role in BD mainly through neuroplasticity changes (Grande et al., 2010). In this regard, genetic links of BDNF to BD are a promising field of research. BDNF Val66Met polymorphism link to BD has been investigated in several studies. The meta‐analysis by Kanazawa et al. (2007) found no association between this polymorphism and BD, whereas Fan and Sklar (2008), in a later meta‐analysis, found a significant association in a more comprehensive review. The most recent analysis by Li et al. (2016) found a significant association between BDNF polymorphism and BD in Europeans unlike the Asians. Thus, the current state of the art suggests that the link between the pathophysiology of BD and BDNF Val66Met polymorphism remains controversial due to heterogeneous findings. In contrast, the findings regarding BDNF peripheral level in patients with BD are more constant. BDNF peripheral level was found to decrease in manic, depressive, and even mixed episodes in several investigations including multiple systematic reviews and meta‐analysis (Fernandes et al., 2011, 2015; Munkholm et al., 2016; Piccinni et al., 2015). Moreover, the stabilization of mood is correlated with the normalization of BDNF peripheral level (Dias et al., 2009), and the factors like psychological stress that lead to a worse course of the disease are associated with a lower level of BDNF (Fernandes et al., 2015; Kapczinski et al., 2008). It is notable that a study by Barbosa et al. (2013) suggests an increased peripheral BDNF level in patients with long‐term BD probably due to antidepressant or mood stabilizer use. Cannabis use is known to associated with earlier age of onset, higher rate of suicide attempts, more disability, and greater risk for mixed episodes (Agrawal et al., 2011; Leite et al., 2015). Among patients with BD, we found a lower peripheral BDNF level in cannabis users in our meta‐analysis. This finding may explain the cannabis use impact on patients with BD.

BDNF level is decreased in the nigrostriatal pathway of patients with PD, and attempts to upregulate BDNF level by gene modulation or direct delivery of BDNF to patients’ brain were unsatisfactory (Howells et al., 2000; Palasz et al., 2020). Furthermore, the severity of cognitive impairment in patients with PD is linked to the peripheral BDNF level (Wang et al., 2016). Additionally, a systematic review by Rahmani et al. found that peripheral BDNF level is decreased in patients with PD (Angelucci et al., 2016). Physical exercise is considered neuroprotective for patients with PD probably due to causing an increase in BDNF level (Angelucci et al., 2016; Palasz et al., 2020; Real et al., 2013). Although some investigations suggest medical use of cannabis in patients with PD (Balash et al., 2017; Venderová et al., 2004), a systematic review by Urbi et al. (2022) did not find certain evidence for cannabis integration into PD treatment despite some potential benefits. Moreover, another systematic review by Thanabalasingam et al. (2021) is in agreement with aforesaid study. Interestingly, our findings also indicate no significant relationship between alternation in BDNF peripheral levels and cannabis use in patients with PD. This may clarify the reasons that patients with PD probably do not benefit from using cannabis.

When interpreting the results based on the past neuropsychiatric history of subjects, caution must be exercised due to the limited number of included studies. It is important to note that neuropsychiatric conditions can independently affect BDNF blood levels. Although the link between cannabis use and NGF peripheral level in our meta‐analysis was nonsignificant, there is evidence suggesting NGF dysregulation in cocaine and heroin users, and moreover, the NGF peripheral level changes are known to happen in a variety of psychological conditions (Angelucci et al., 2007; Chen et al., 2015; Pedrotti Moreira et al., 2019).

Onrell et al.’s systematic review and meta‐analysis which investigated BDNF level and substance use disorders found a relationship between the dysregulation of BDNF and using alcohol or crack‐cocaine, and a possible relationship between BDNF peripheral levels and the severity of drug dependence. Moreover, they found a general decrease in peripheral BDNF levels of drug users by pooling the data from several types of drug users, in contrast to our findings regarding BDNF peripheral levels and cannabis use (Ornell et al., 2018). Our review is strengthened by the development of a comprehensive systematic search protocol for obtaining up‐to‐date results from four databases, increasing the accuracy and reliability of our conclusions. In addition, we checked the supplementary data of studies in cases where the required data was not disclosed in the full article text, and we analyzed and assessed the confidence interval of each outcome.

Our study faced limitations. Differences in the duration of cannabis use and severity of dependence of subjects among included studies and the use of concomitant treatments such as antipsychotics or antidepressants, which may cause false attribution of some outcomes to cannabis. Moreover, we acknowledge that the absence of subjects with a definitive diagnosis of cannabis use disorder may limit the strength of our findings, as individuals with more severe dependence may exhibit more prominent symptoms and alterations in BDNF blood levels. Additionally, it is worth noting that previous research has indicated that the consumption of alcohol, psychedelics, and tobacco can also disrupt BDNF expression (Colle et al., 2016; Hutten et al., 2020; Silva‐Peña et al., 2019), which is relevant in the context of cannabis users who may use other substances as well (Fergusson & Horwood, 2000; Fergusson et al., 2006). This may have an impact on our findings and should be considered another limitation of our study. Thus, several aspects of heterogenicity are noted in our study. It is critical to keep in mind that when evaluating a factor such as BDNF that has different levels in males and females, the resulting outcomes may differ between genders, potentially influencing the reliability of our findings. Hence, additional studies with higher levels of evidence are needed to investigate cannabis link with neurotrophic factors more accurately, and caution should be exercised when interpreting our results.

To conclude, this study indicates that there is no significant relationship between cannabis use and BDNF or NGF peripheral levels. The lack of association may be due to various factors, such as the neuropsychiatric history and medication use of the participants. As the available data is limited and the findings uncertain, we encourage further investigations, particularly longitudinal studies that account for the neuropsychiatric history of cannabis users.

## AUTHOR CONTRIBUTIONS

**Arman Shafiee**: Conceptualization; project administration; data curation; writing—original draft; writing—review and editing; visualization. **Mahmood Bakhtiyari**: Validation; resources; methodology; software; formal analysis; writing—original draft. **Mohammad Ali Rafiei**: Validation; resources; methodology; software; formal analysis; writing—original draft. **Mohammad Javad Amini**: Validation; resources; methodology; software; formal analysis; writing—original draft. **Arman Shafiee**: Validation; resources; methodology; software; formal analysis; writing—original draft. . **Alireza Eskandari**: Writing—original draft. **Kyana Jafarabady and Faeze Soltani Abhari**: Data curation; project administration.

## CONFLICT OF INTEREST STATEMENT

The authors have no relevant affiliations or financial involvement with any organization or entity with a financial interest in or financial conflict with the subject matter or materials discussed in the manuscript.

## FUNDING INFORMATION

This study did not receive funding, grant, or sponsorship from any individuals or organizations.

### PEER REVIEW

The peer review history for this article is available at https://publons.com/publon/10.1002/brb3.3340.

## Supporting information



Supporting Information

## Data Availability

All data has been presented in the manuscript.
